# Ventricular fibrillation triggered by cavotricuspid isthmus radiofrequency ablation with a dual-energy lattice-tip catheter in a patient with an implantable cardioverter-defibrillator

**DOI:** 10.1016/j.hrcr.2025.11.004

**Published:** 2025-11-10

**Authors:** Celina V. Malyar, Bakhtawar Khan Mahmoodi, Sing-Chien Yap

**Affiliations:** Department of Cardiology, Thorax Center, Cardiovascular Institute, Erasmus MC, Rotterdam, The Netherlands

**Keywords:** Lattice-tip catheter, Current shunting, Implantable cardioverter-defibrillator, Ventricular fibrillation, Atrial flutter

**Key Teaching Points**

• Radiofrequency (RF) ablation near an implantable cardioverter-defibrillator (ICD) lead can induce current, leading to myocardial capture at the lead tip and, in rare cases, triggering ventricular fibrillation.
• Cavotricuspid isthmus RF ablation with a dual-energy lattice-tip catheter should be performed cautiously in patients with transvenous ICDs because of the risk of lead–device interaction.
• Clinical data on the safety of the novel lattice-tip catheter in patients with ICDs remain limited.

## Introduction

Radiofrequency (RF) ablation of the cavotricuspid isthmus (CTI) is an effective and widely used treatment for typical atrial flutter. However, performing RF ablation in patients with cardiac implantable electronic devices, such as implantable cardioverter-defibrillators (ICDs), introduces safety considerations.

One of the most important concerns is the interaction between RF energy and transvenous leads. When ablation is performed near an ICD lead, RF current can be shunted through the metallic components of the lead. This may result in electromagnetic interference (EMI), device malfunction, lead damage, tissue injury, and, in rare instances, ventricular arrhythmias by direct myocardial capture.^1^^,^^2^ Although novel ablation tools such as the lattice-tip ablation catheter have been designed to optimize lesion delivery and safety, clinical data on their use in patients with ICDs remain limited.^3^^,^^4^

We report a case of ventricular fibrillation (VF) induced during CTI RF ablation using a lattice-tip ablation catheter in a patient with a transvenous ICD. This case highlights the need for heightened awareness and caution when performing RF ablation near ICD leads and emphasizes the importance of preventive strategies and readiness for prompt arrhythmia management.

## Case report

We present a 56-year-old man with symptomatic paroxysmal regular atrial tachyarrhythmia and surgically corrected tetralogy of Fallot who was referred to our center for a second redo ablation. He had a medical history of pulmonary valve replacement, surgical atrial septal closure, dual-chamber ICD implantation (secondary prevention), and 2 previous catheter ablations for paroxysmal regular atrial tachyarrhythmia (before ICD implantation). During his first catheter ablation in 2011, a macroreentry atrial tachycardia (AT) could be induced, which was related to an incisional scar on the lateral right atrial wall. The tachycardia was successfully treated by a bicaval ablation line over the incisional scar. Owing to medically refractory recurrent symptomatic paroxysmal regular AT, he underwent a redo ablation procedure in 2020. During the second procedure, a CTI-dependent macroreentry AT could be induced and was successfully treated with a CTI ablation. 5 years after the second procedure, he again presented with symptomatic paroxysmal regular AT, and he was scheduled for a redo procedure using the Affera mapping (Prism-1) and ablation (Sphere-9) system (Medtronic). The Sphere-9 is an 8F catheter with a 9-mm compressible nitinol lattice tip, containing 9 minielectrodes on the spherical surface, which can deliver both RF and pulsed field energy.^3^ Temperature-controlled RF energy is delivered through the entire conductive lattice tip, whereas saline is evenly dispersed from a central irrigation nozzle at a rate of 30 mL/min during RF application.

**Figure 1:**
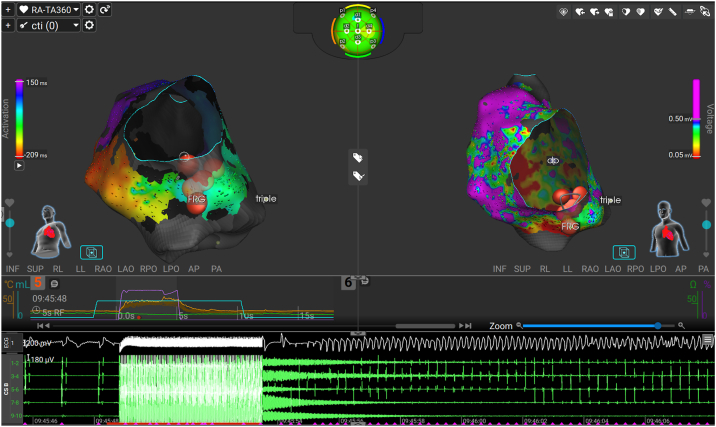
3-dimensional activation and voltage mapping. The left panel shows the activation mapping of the counterclockwise peritricuspid atrial flutter. The whole tachycardia cycle length could be mapped. The right panel demonstrates the close unipolar voltage map with a voltage cutoff of 0.5 mV. The *red ablation tags* mark ablation points on the cavotricuspid isthmus. The ablation graph demonstrates the surface temperature (*orange graph*), irrigation flow rate (*blue graph*), and current output (*purple graph*) of the third consolidation radiofrequency application inducing ventricular fibrillation. There was no excessive temperature rise. At the bottom, the electrocardiogram and intracardiac coronary sinus signals are displayed. Notice ventricular fibrillation directly after termination of radiofrequency energy.

**Figure 2:**
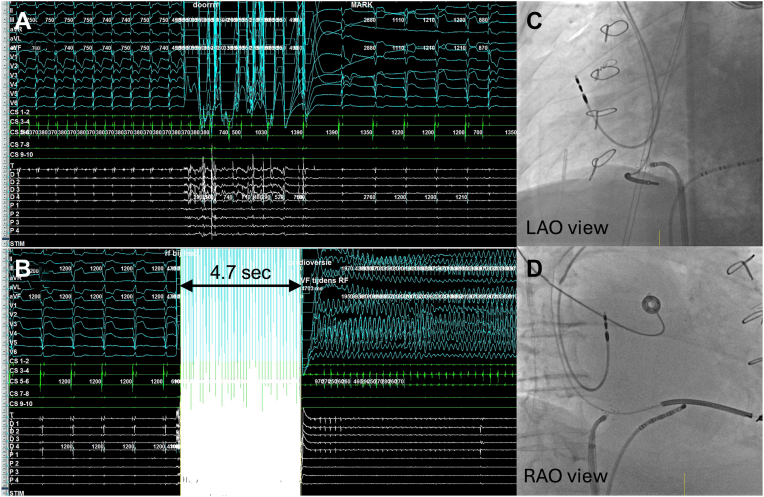
**A:** intracardiac tracing during the first radiofrequency (RF) application, which terminated the atrial flutter. The surface electrocardiogram (ECG) (*blue*), CS signals (*green*), and Sphere-9 signals (*white*) are depicted. Please note the artifacts on the Sphere-9 and surface ECG during RF ablation. **B:** Intracardiac tracing during the third consolidation RF application, which induced ventricular fibrillation. Prior to ventricular fibrillation induction, signal oversaturation is observed on the electrode signals of the Sphere-9 ablation catheter and surface ECG during RF energy delivery for 4.7 seconds. **C and D:** fluoroscopy images in LAO and RAO projection demonstrating that the ablation catheter was in contact with the implantable cardioverter-defibrillator lead (proximal to the implantable cardioverter-defibrillator coil) during the third consolidation RF application. LAO = left anterior oblique; RAO = right anterior oblique.

The procedure was performed under general anesthesia. The ICD was deactivated at the start of the procedure to minimize the risk of inappropriate shock (Biotronik Acticor 7 DR-T; Solia S53 atrial lead; Pamira S65 ICD lead). 3-dimensional mapping during pacing from the coronary sinus demonstrated a bicaval ablation line with conduction block and reconduction over the previous CTI ablation line. Furthermore, there were extensive areas of low-voltage zones (<0.5 mV) in the right atrium. Using atrial burst pacing, a counterclockwise CTI-dependent macroreentry AT (cycle length 370 ms) could be induced (Figure 1). It is advised to use RF energy for CTI ablation with the following settings: 5 seconds duration, target surface temperature 73°C, current limit 80%, irrigation rate 30 mL/min). The tachycardia terminated during the first RF application with the Sphere-9 catheter at the critical isthmus on the CTI (Figure 2A). During this application, there were small artifacts on the intracardiac signals of the Sphere-9 and the surface electrocardiogram (ECG). To create a durable CTI line, 3 additional overlapping consolidation RF applications were subsequently placed around the initial lesion. During the third consolidation RF application at the CTI-inferior vena cava junction, the patient developed VF (Figure 2B). During the RF application, there were large artifacts on the intracardiac signals of the Sphere-9 and the surface ECG. Fluoroscopic imaging directly after the event demonstrated that the ablation catheter was in close contact with the ICD lead (proximal to the coil) at the time of VF induction (Figure 2C and 2D). The patient was successfully defibrillated to sinus rhythm using an external defibrillator. There were no ST-segment abnormalities after ablation suggestive of the occurrence of coronary spasm. After achieving hemodynamic stability, the CTI block was confirmed after a 25-minute waiting period.

Postablation ICD interrogation did not show any significant change in parameters in comparison with baseline values (Table 1). Furthermore, no episodes were documented by the ICD. The patient had an uncomplicated postablation observation period and could be discharged from the hospital the same day. ICD interrogation 1 month after the procedure demonstrated no significant change in parameters (Table 1). There was no recurrence of any AT during the short-term follow-up of 3 months.

**Table 1:** ICD interrogation before and after ablation

Parameter	Before ablation	After ablation	Follow-up after 1 mo
Atrial lead			
Sensing	1.3 mV	1.2 mV	0.6 mV
Threshold	0.5 V/0.4 ms	0.5 V/0.4 ms	0.5 V/0.4 ms
Impedance	660 Ω	464 Ω	601 Ω
Ventricular lead			
Sensing	19.3 mV	18.1 mV	18.5 mV
Threshold	0.5 V/0.4 ms	0.4 V/0.4 ms	0.5 V/0.4 ms
Impedance	504 Ω	464 Ω	504 Ω
Shock impedance	50 Ω	46 Ω	53 Ω

ICD = implantable cardioverter-defibrillator.

## Discussion

When RF energy is applied near a transvenous ICD or pacemaker lead, several problems may occur including remote heating of the lead tip (ohmic heating), alteration of the lead-tissue interface, EMI, lead insulation damage, device damage, and lead dislodgement.^5^ During ohmic heating, the lead acts as a conductor, concentrating RF energy, which leads to heating of the lead tip and surrounding myocardium.^6^ This may result in increased capture threshold, reduced sensing, and tissue damage.^2^ EMI during RF ablation can lead to transient oversensing, undersensing, pacing inhibition, inappropriate shocks, or inappropriate pacing behavior, possibly causing ventricular arrhythmia. Modern pulse generators are less sensitive to EMI. Rarely, lead revision or generator replacement is necessary when there is a permanent lead or device malfunction after ablation.

The risk of inadvertent effects increases with closer proximity of the RF ablation catheter to the ICD or pacemaker lead (tip). For example, the incidence of lead or device malfunction is lower in patients undergoing atrial fibrillation ablation than those undergoing atrioventricular junction ablation.^2^^,^^7^ To reduce risk, several precautions can be taken such as avoiding RF energy close to the lead tip or lead body; monitoring device function before, during, and after ablation (ie, sensing, capture thresholds, lead impedance); disable antitachycardia therapy to prevent inappropriate shocks; adjust pacing modes in pacemaker-dependent patients; and waiting at least 6 weeks after implantation to reduce the risk of lead dislodgement.^1^

Besides the proximity of the ablation catheter to the transvenous lead, our case demonstrates that a larger effective surface area of the ablation tip may potentially play a role in the adverse device effects. The lattice tip serves as a continuous conductive ablation electrode with an effective surface area 10× larger (275 mm^2^) than standard 3.5–4 mm tip irrigated ablation catheters (27–31 mm^2^), which results in lower current density and thus allows higher current (approximately 560 W at 50 Ω) without the risk of a steam pop.^8^ However, the larger surface area in combination with high current may increase the risk of conducting RF energy to the lead tip when ablating close to the transvenous lead. There are several potential explanations for VF induction during RF ablation on a transvenous lead. Lead tip heating can occur through the induction of eddy currents during monopolar RF ablation when the ICD lead acts as a secondary antenna, thus inducing ventricular arrhythmia. The artifacts on the electrodes and surface ECG are highly suggestive of close contact of the Sphere-9 with metallic components of the ICD lead. However, a previous study demonstrated that the risk of insulation damage during RF ablation on the body of a transvenous lead is small.^9^ Another explanation is a R-on-T phenomenon secondary owing to undersensing or noise reversion mode. An inappropriate pacing stimulus coinciding with the T-wave may trigger VF.

Unexpected VF induction during ablation with the Affera system has been described in another case when pulsed field energy was used to create a mitral isthmus line in a patient with a dual-chamber pacemaker.^10^ To the best of our knowledge, our case is the first report of VF induction during RF ablation on the CTI with the Affera system in a patient with an ICD. This case highlights the need for caution when performing CTI ablation with the Affera system in patients with a pacemaker or ICD.

## Conclusion

This case, to the best of our knowledge, is the first reported instance of VF induction during CTI RF ablation with a lattice-tip ablation catheter in a patient with an ICD. The VF was likely triggered by conduction of RF energy through the ICD lead, highlighting the need for caution when performing CTI ablation in such patients.

## Disclosures

S.C.Y. has received research funding from Medtronic, Biotronik, Boston Scientific, and Biosense Webster. In addition, he has received honoraria from Boston Scientific and Biosense Webster. The other authors have no conflicts of interest to disclose.
